# Radiology Education Among Emergency Medicine Residencies: A National Needs Assessment

**DOI:** 10.5811/westjem.2021.6.52470

**Published:** 2021-09-02

**Authors:** Stephen E. Villa, Natasha Wheaton, Steven Lai, Jaime Jordan

**Affiliations:** University of California, Los Angeles, David Geffen School of Medicine, Department of Emergency Medicine, Los Angeles, California

## Abstract

**Introduction:**

Radiology training is an important component of emergency medicine (EM) education, but its delivery has been variable. Program directors have reported a lack of radiology skills in incoming interns. A needs assessment is a crucial first step toward improving radiology education among EM residencies. Our objective was to explore the current state of radiology education in EM residency programs.

**Methods:**

This was a cross-sectional survey study of all Accreditation Council for Graduate Medical Education-accredited EM programs in the United States. Program leadership completed an online survey consisting of multiple choice, Likert scale, and free-response items. We calculated and reported descriptive statistics.

**Results:**

Of eligible EM programs, 142/252 (56%) completed the survey including 105 postgraduate year (PGY) 1–3 and 36 PGY 1–4 programs. One respondent opted out of answering demographic questions. 23/141 (16%) were from the Western region, 29/141 (21%) were from the North Central region, 14/141 (10%) were from the South-Central region, 28/141 (20%) were from the Southeast region, and 47/141 (33%) were from the Northeast region. A total of 88/142 (62%) of responding programs did not have formal radiology instruction. Of the education that is provided, 127/142 (89%) provide it via didactics/lectures and 115/142 (81%) rely on instruction during clinical shifts. Only 51/142 (36%) provide asynchronous opportunities, and 23/142 (16%) have a dedicated radiology rotation. The majority of respondents reported spending 0–2 hours per month on radiology instruction (108/142; 76%); 95/141 (67%) reported that EM faculty “often” or “always” provide radiology instruction; 134/142 (95%), felt that it was “extremely” or “very important” for ED providers to be able to independently interpret radiograph results; and 129/142 (90.84%) either “sometimes” or “always” rely on their independent radiograph interpretations to make clinical decisions. The radiology studies identified as most important to be able to independently interpret were radiographs obtained for lines/tubes, chest radiographs, and radiographs obtained for musculoskeletal-related complaints.

**Conclusion:**

A minority of EM residency programs have formal instruction in radiology despite the majority of responding program leadership believing that these are important skills. The most important curricular areas were identified. These results may inform the development of formal radiology curricula in EM graduate medical education.

## INTRODUCTION

In the acute setting, rapid and accurate interpretation of diagnostic imaging is critical to patient care, especially in clinical arenas that require real-time interpretation such as the emergency department (ED). Studies have also shown attending radiologist coverage is variably available, necessitating emergency physicians to make treatment decisions based on their own interpretation.^1^ Prior literature has shown wide variability in radiologist and emergency provider concordance with respect to interpretations of studies, which raises the question of accuracy of interpretation by emergency physicians.^2^–^8^ This may be due to inadequate training for such tasks. Radiology instruction is variable in undergraduate and graduate medical education, ranging from informal teaching to required educational experiences.^9^,^10^ This variability in exposure and training may lead to varying provider competency. In fact, a recent survey of emergency medicine (EM) attendings found that only 30% felt prepared to independently interpret plain films on their own at graduation from their residency.^10^

In 2015, members of the Society of Academic Emergency Medicine (SAEM) along with members from several radiology organizations met and agreed that the ability to select and interpret diagnostic imaging is an integral skill for EM providers and, therefore, recommended that a diagnostic imaging curriculum and tools to assess competency aimed at EM residency training be developed.^11^ It is unclear to what extent these recommendations have been implemented. As it stands, no standardized nationwide radiology curriculum aimed at EM residents exists. A national needs assessment of education leaders within our specialty is an important first step to developing optimal curricula in radiology for EM residents. In this study we aimed to explore the current state of radiology instruction in EM residency programs in the United States and to identify priorities for future curricula.

## METHODS

### Study Setting and Participants

We identified US EM programs accredited by the Accreditation Council for Graduate Medical Education (ACGME) through the ACGME website in March 2020.^12^ We invited one member of the program leadership from each program to participate based on available contact information, with preference for program director over assistant/associate program director over medical student directors. We collected data between March–September 2020. This study was deemed exempt by the institutional review board of University of California, Los Angeles.

### Study Design

This was a cross-sectional survey study. We identified contact information for potential participants through the ACGME website, the SAEM residency directory,^13^ programs’ individual websites, and study team members’ personal knowledge. We invited subjects to participate by email and provided them with a link to an internet-based survey administered through SurveyMonkey.^14^ We sent two follow-up email invitations at weekly intervals to non-responders. Informed consent was implied by those who chose to complete the survey.

**Population Health Research Capsule**

What do we already know about this issue?

*In 2015 members of the Society of Academic Emergency Medicine and other organizations recommended that radiology curricula to assess competency be developed*.

What was the research question?

*What is the current state of radiology education among emergency medicine residency programs?*

What was the major finding of the study?

*A minority of programs have formal instruction despite program leadership believing it is important*.

How does this improve population health?

*Understanding the current state of radiology education lays the foundation for improving radiology instruction, hopefully leading to better care for patients*.

### Instrument

One author with advanced training in survey design (SV) developed the survey after literature review and input from other expert EM educators to maximize content validity. The survey consisted of Likert scale, multiple choice, and free-response items. The survey was read aloud and discussed among members of the study group and piloted with a small group of representative subjects to ensure response process validity. We made revisions for clarity and readability. To maximize response rate and minimize guessing on items that participants didn’t feel able to answer, respondents were not required to complete every question. The final version of the survey is available in Appendix A.

### Data Analysis

We calculated and reported descriptive statistics for multiple choice and Likert items. We performed a thematic analysis of data from the single free-response item.

## RESULTS

We identified contact information for 252 ACGME-accredited EM programs. A total of 142 (56.35%) completed the survey. Characteristics of participating programs are shown in Table 1.

More than half, 88/142 (61.97%), of EM programs did not have formal instruction in radiology. Programs provide instruction through didactics/lectures (127/142, 89.44%), instruction during clinical shifts (115/142, 80.99%), and asynchronous education (23/142, 16.20%). Just 23 programs (16.20%) have a dedicated radiology rotation. When given the opportunity to elaborate on their responses through free text, 16 respondents offered other unique areas where radiology education was provided to their residents, which included ultrasound rotations (eight respondents), radiology electives (six respondents), orthopedics rotations (one respondent) and anesthesia rotations (one respondent).

Programs dedicated varying amounts of time to radiology instruction outside of clinical shifts with the most common (108/142; 76.06%) being 0–2 hours per month. Four programs (2.82%) provided no instruction outside of clinical shifts. Twenty-one programs (14.97%) spent more than two hours but not more than four hours per month, seven programs (4.93%) spent more than four but not more than six hours per month, one program (0.70%) spent more than six but not more than eight hours per month, one program (0.70%) spent more than eight but not more than 10 hours per month, and no programs spent more than 10 hours per month.

Emergency medicine faculty were the instructors most commonly providing instruction in radiology to EM residents with 95/141 (67.38%) programs indicating that this group either “always” or “often” provided instruction. Of 138 programs, 60 (43.48%) indicated that EM residents (including self-study) either “always” or “often” provided instruction. Radiology faculty were noted to “sometimes” (47/137, 34.31%) or “rarely” (49/137, 35.77%) provide instruction. Radiology residents “sometimes” (20/139, 14.39%) or “rarely” (31/139, 22.3%) provided instruction. Other faculty/residents noted to provide instruction included the following: neurology; sports medicine/orthopedics; obstetrics & gynecology; and surgery. See Table 2.

The majority (134/142; 95.03%) of respondents felt that it was “extremely” or “very important” for ED providers to be able to independently interpret radiograph results. Sixty-eight of 142 (48.22%) felt it was “extremely” or “very important” for ED providers to independently interpret computed tomography (CT) images. See Figure and Table 3. Seventeen leaders responded with “It depends” for the importance of independent CT interpretation, with 12 commenting that CT head is more important than other types of CT. Additional free-text responses commented on the wording of “independently interpret,” elaborating that they expect residents to be familiar with but not experts in CT interpretations. With respect to magnetic resonance imaging (MRI), the majority of the respondents (87/142; 61.27%) stated it was “not at all important” or “not so important” for emergency care providers to be able to independently interpret those studies.

Almost 9% (12/142) of respondents “always” relied on their own radiograph interpretation, while 52 respondents (36.6%, 52/142) “usually” relied on their own radiograph interpretation and 45.8% (65/142) “sometimes” relied on their own interpretation. With respect to CT, 1% (2/141) “always” relied on their own interpretation. Eight percent (12/141) “usually” relied on their own CT interpretation, and 42% (59/141) “sometimes” relied on their own interpretation. Regarding availability of attending radiology coverage, only half of responding programs (73/141, 51.77%) indicated that this was “always” available with 37.59% (53/141) noting it was “usually” and 10.64% (15/141) “sometimes” available. No programs reported that attending radiology coverage was “rarely” or “never” available.

The most common radiology studies that respondents believed residents should be able to interpret independently at graduation were radiographs obtained for lines/tubes, chest radiographs and radiographs obtained for musculoskeletal-related complaints (Table 4).

Twenty-six participants provided additional free-text comments at the end of the survey. One major theme that emerged was the importance of being able to detect emergent, time-sensitive pathology. For example, one respondent commented: “the EM resident’s review [should] focus on identifying major abnormalities for the modality, intracranial hemorrhage (ICH) on head CT, appendicitis on CT abdomen/pelvis, etc.” Another major theme was the expectation of basic familiarity, but not expertise, with imaging interpretation. As one respondent aptly put it: “basic radiology should be expected and … tested by ABEM [American Board of Emergency Medicine] for certification, complex reads should not be expected.” Lastly, respondents highlighted the need for EM radiology curricula. Exemplar quotes include the following:

*“We use several, albeit woefully lacking for our needs, websites for instruction. We are exploring creation of our own site.” “I have looked for some sort of turn-key EM resident radiology curriculum but have yet to find anything suitable. This is where the specialty of EM needs to come together to make a nationwide curriculum to teach our trainees what they need to know.”*

## DISCUSSION

Our study of EM education leaders demonstrates that a large number of residency programs do not have a formalized radiology curriculum despite respondents feeling that providers should be able to interpret many studies independently. Most programs in this study rely first on EM faculty followed by EM residents followed by other specialties for their radiology instruction. Our study also demonstrates that a variety of methods are being used to provide this education, which is likely somewhat reflective of the available resources at various institutions. Despite calls for formalizing a radiology curriculum in 2015,^11^ it appears that many programs have yet to achieve this goal. Currently, most programs deliver radiology curricula via didactics and on-shift teaching. While prior literature has demonstrated that confidence of radiology interpretation skills of recent graduates can be improved by on shift teaching, this clinical education may be of variable quality and quantity depending on the individual training program.^11^ This is supported by literature demonstrating that EM attendings’ confidence in their own radiology interpretation skills is affected by the type of program they trained at as well as whether they were required to independently interpret studies during residency.^11^

Our study found that the vast majority of programs dedicate less than four hours per month to radiology-related concepts, and without a structured educational plan including specific goals and objectives this training may be inadequate to prepare residents for future job tasks. Our findings support the call from Gunn et al for the creation of formalized curricula and tools to assess competency in this area.^11^ Finally, while asynchronous learning opportunities in radiology are available, our study highlights that many programs are not capitalizing on this additional teaching modality, despite some programs and prior studies demonstrating success with use of this modality.^15^,^16^

Many institutions in our study rely on their own interpretations, specifically for radiographs. This is in accordance with prior literature that has demonstrated attending radiology coverage is variable.^1^,^17^,^18^ Our results suggest that it is more common for emergency physicians to rely on their own interpretations of radiographs as compared to CT images, which may highlight why respondents felt that it was more important for graduating residents to be able to independently interpret radiographs as compared to CT. This emphasizes that radiograph interpretation should be a focus in future EM radiology curricula. While radiograph interpretation skills are essential, many respondents in our study also pointed out the importance of the ability to assess for critical, time-sensitive pathology on CT. For example, rapid interpretation of CT head and reassurance that it is negative for ICH is necessary for the decision to push tissue plasminogen activator (tPA)in suspected stroke.^19^ While hospitals may have a board-certified radiologist available for the interpretation of CT, many institutions use tele-radiology overnight and on weekends,^1^,^17^ and not all institutions have nighttime CT images read in time for patient care decisions.^17^ It is, therefore, necessary that future EM radiology curricula include education on how to assess for time-sensitive emergent pathologies on CT.

More specifically, our results highlight that select imaging studies are seen as important for graduating residents to be able to independently interpret, which should further inform curricular development. While it would be ideal to provide a foundational understanding for all studies ordered in the ED, our findings demonstrate future radiology curricula should prioritize teaching interpretation of radiographs obtained for lines/tubes, chest radiographss and radiographs obtained for musculoskeletal related complaints, followed by specific CT studies, primarily CT head. These specific studies are in line with the time pressure of making a rapid decision affecting patient care (ie, pushing tPA for possible stroke or adjusting an endotracheal tube for a patient who was recently intubated, or whether a central line is suitable for use]). This time pressure coupled with the reality that ED providers are likely to be making interpretations independently therefore reinforces that these specific areas should be prioritized.

Further comparative studies are needed to understand which methods or combination of methods are most effective for delivering this core content. While many curricula have focused on knowledge and skills with respect to interpretation, it may also be important to include other facets related to radiology, such as appropriateness of obtaining studies, associated risks, and cost/benefit assessments.^20^,^21^,^22^ We are hopeful that our results help inform the development of future radiology curricula for EM residents.

## LIMITATIONS

This was a survey study, and the results must be considered within the limitations of this type of design. Despite collecting data from a large number of programs from diverse locations, institution types and program formats, we were not able to obtain data from all programs, which may limit the generalizability of our results. Another limitation is that we purposefully did not ask respondents about ultrasound, a commonly performed and ordered study in the ED. Given emergency ultrasound is recognized by the ACGME and the American Board of Emergency Medicine as a core competency and is a required milestone for graduates, many programs likely have dedicated curriculums to achieve competency for point-of-care ultrasound (POCUS). Given that other studies have characterized competency and needs in ultrasound teaching, we chose not to include ultrasound as a modality in our study to reduce confusion between radiology-assisted (or “formal”) ultrasound and POCUS.^23^,^24^

## CONCLUSION

A minority of EM residency programs in our study reported having formal training in radiology despite the majority of program leadership believing that these are important skills for residents to develop during training. The most important curricular areas were predominantly radiographs. These results should inform the development of formal radiology curricula within emergency medicine.

## Supplementary Information



## Figures and Tables

**Figure:**
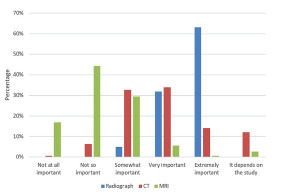
Perceived importance of independent interpretation of radiology studies. *CT*, computed tomography; *MRI*, magnetic resonance imaging.

**Table 1:** Characteristics of emergency medicine residency programs.

	N* (% of total)
Program Format	
PGY 1–3 years	105 (74.47%)
PGY 1–4 years	36 (25.53%)
Primary Clinical Site	
County	21 (14.89%)
University	58 (41.13%)
Community	54 (38.30%)
Other	8 (5.67%)
Program Region	
Western Region (AK, AZ, CA, CO, HI, ID, MT, NM, NV, OR, UT, WA, WY)	23 (16.31%)
North Central Region (IA, IL, IN, MI, MN, ND, NE, OH, SD, WI)	29 (20.57%)
South Central Region (AR, KS, LA, MO, OK, TX)	14 (9.93%)
Southeast Region (AL, FL, GA, KY, MS, NC, PR, SC, TN, VA, VI, WV)	28 (19.86%)
Northeast Region (CT, DC, DE, MA, MD, ME, NH, NJ, NY, PA, RI, VT)	47 (33.33%)

* 1 respondent opted out of the demographic portion of the survey leaving 141 responses out of 142 responses.

*PGY*, postgraduate year.

**Table 2:** Personnel providing radiology instruction to emergency medicine residents.

	Never N (%)	Rarely N (%)	Sometimes N (%)	Often N (%)	Always N (%)	Total N*
Group						
EM faculty	1 (0.71%)	6 (4.26%)	39 (27.66%)	69 (48.94%)	26 (18.44%)	141
EM residents (includes self-study)	2 (1.45%)	12 (8.70%)	64 (46.38%)	50 (36.23%)	10 (7.25%)	138
Radiology faculty	25 (18.25%)	49 (35.77%)	47 (34.31%)	12 (8.76%)	4 (2.92%)	137
Radiology residents	84 (60.43%)	31 (22.30%)	20 (14.39%)	3 (2.16%)	1 (0.72%)	139
Other specialty faculty	43 (32.33%)	46 (34.59%)	37 (27.82%)	7 (5.26%)	0 (0%)	133
Other specialty residents	75 (57.69%)	31 (23.85%)	22 (16.92%)	2 (1.54%)	0 (0%)	130

* Note, some questions were skipped by respondents.

*EM*, emergency medicine.

**Table 3:** Perceived importance of emergency care providers’ ability to independently interpret different radiology studies.

	Not at all important N (%)	Not so important N (%)	Somewhat important N (%)	Very important N (%)	Extremely important N (%)	It depends on the study N (%)	N*
Study type							
Radiograph	0 (0%)	0 (0%)	7 (4.96%)	45 (31.91%)	89 (63.12%)	0 (0%)	141
CT	1 (0.71%)	9 (6.38%)	46 (32.62%)	48 (34.04%)	20 (14.18%)	17 (12.06%)	141
MRI	24 (16.90%)	63 (44.37%)	42 (29.58%)	8 (5.63%)	1 (0.7%)	4 (2.82%)	142

* Note: 1 respondent skipped questions specific to radiograph and CT.

*CT*, computed tomography; *MRI*, magnetic resonance imaging.

**Table 4:** Percentage of agreement with the following statement: “Residents should be able to independently interpret the following radiology study at graduation.”

	Strongly disagree N (%)	Disagree N (%)	Neutral N (%)	Agree N (%)	Strongly agree N (%)	Total N*
Radiograph for line or tube placement (central line, ET tube, NG/G tube)	2 (1.41%)	0 (0.00%)	0 (0.00%)	5 (3.52%)	135 (95.07%)	142
Chest radiograph	2 (1.42%)	0 (0.00%)	1 (0.71%)	5 (3.55%)	133 (94.33%)	141
MSK radiograph (ie, shoulder, elbow, wrist, hand, knee, ankle, foot, etc.)	2 (1.41%)	0 (0.00%)	3 (2.11%)	23 (16.20%)	114 (80.28)	142
Pelvis radiograph	2 (1.43%)	0 (0.00%)	6 (4.29%)	20 (14.29%)	112 (80.00%)	140
Soft tissue neck radiograph (ie, pediatric stridor)	2 (1.41%)	2 (1.41%)	16 (11.27%)	42 (29.58%)	80 (56.34%)	142
CT brain (non-contrast)	1 (0.70%)	5 (3.52%)	10 (7.04%)	54 (38.03%)	72 (50.70%)	142
Abdominal radiograph	2 (1.42%)	1 (0.71%)	22 (15.60%)	47 (33.33%)	69 (48.94%)	141
CT cervical spine	2 (1.42%)	17 (12.06%)	43 (30.50%)	52 (36.88%)	27 (19.15%)	141
CT abdomen/pelvis	3 (2.11%)	19 (13.38%)	42 (29.58%)	62 (43.66%)	16 (11.27%)	142
CT angiography chest (ie, PE)	5 (3.52%)	23 (16.20%)	48 (33.80%)	52 (36.62%)	14 (9.86%)	142
CT chest	7 (4.93%)	21 (14.79%)	56 (39.44%)	50 (35.21%)	8 (5.63%)	142
CT extremity	15 (10.56%)	45 (31.69%)	55 (38.73%)	20 (14.08%)	7 (4.93%)	142
CT/CT angiography (ie, stroke protocol)	15 (10.56%)	45 (31.69%)	52 (36.62%)	27 (19.01%)	3 (2.11%)	142
MRI brain	40 (28.17%)	49(34.51%)	36 (25.35%)	16 (11.27%)	1 (0.70%)	142
MRI spine	43 (30.28%)	50(35.21%)	33 (23.24%)	15 (10.56%)	1 (0.70%)	142

* Note, some questions were skipped by respondents.

*ET*, endotracheal; *NG*, nasogastric tube; *G*, gastric; *MSK*, musculoskeletal; *CT*, computed tomography; *MRI*, magnetic resonance imaging; *PE*, pulmonary embolism.
