# Supplementary material for: Radiology Education Among Emergency Medicine Residencies: A National Needs Assessment
Source: West J Emerg Med. 2021 Sep 2;22(5):1110–6. doi: 10.5811/westjem.2021.6.52470 (PMC8463068; doi:10.5811/westjem.2021.6.52470)
Supplement: Supplementary file 1 [file wjem-22-1110-s001.docx]

Radiology Needs Assessment

Appendix A: Survey:

1. Do you have a formalized radiology curriculum in your residency program?
   1. Yes/No
2. How does your program provide radiology instruction to residents? (select all that apply)
   1. Didactic lectures
   2. Asynchronous online instruction
   3. Instruction during clinical shifts
   4. Dedicated radiology rotation
   5. Other off service rotations
      1. If selected Other: Please describe
3. Outside of clinical shifts, approximately how much time is devoted to radiology education?
   1. None
   2. 0-2 hours per month
   3. >2-4 hours per month
   4. >4-6 hours per month
   5. >6-8 hours per month
   6. >8-10 hours per month
   7. >10 hours per month
4. How often do the following groups of people provide instruction to your residents in radiology?

|  | Never | Rarely | Sometimes | Often | Always |
| --- | --- | --- | --- | --- | --- |
| EM Faculty |  |  |  |  |  |
| EM residents (includes self-study) |  |  |  |  |  |
| Radiology faculty |  |  |  |  |  |
| Radiology residents |  |  |  |  |  |
| Other specialty faculty |  |  |  |  |  |
| Other specialty residents |  |  |  |  |  |

- 1. If select “Other”: Please specify: Free Text Response

1. How important is it for ED providers (including residents) to be able to independently interpret x-rays ordered in the Emergency Department?
   1. Not at all important
   2. Not so important
   3. Somewhat important
   4. Very important
   5. Extremely important
   6. “It depends on the x-ray”: Please elaborate: Free Text Response
2. How important is it for ED providers (including residents) to be able to independently interpret CT Scans ordered in the Emergency Department?
   1. Not at all important
   2. Not so important
   3. Somewhat important
   4. Very important
   5. Extremely important
   6. “It depends on the x-ray”: Please elaborate: Free Text Response
3. How important is it for ED providers (including residents) to be able to independently interpret MRI’s ordered in the Emergency Department?
   1. Not at all important
   2. Not so important
   3. Somewhat important
   4. Very important
   5. Extremely important
   6. “It depends on the x-ray”: Please elaborate: Free Text Response
4. Overall, in your practice, how frequently do you or your residents rely on your own interpretation of x-rays to make treatment decisions, prior to the official report?
   1. Never
   2. Rarely
   3. Sometimes
   4. Usually
   5. Always
5. Overall, in your practice, how frequently do you or your residents rely on your own interpretation of CT scans to make treatment decisions, prior to the official report?
   1. Never
   2. Rarely
   3. Sometimes
   4. Usually
   5. Always
6. Please rate your agreement with the following statement. Residents should be able to independently interpret the following radiologic studies at graduation. (1 = strongly disagree, 5 = strongly agree)

|  | Strongly Disagree | Disagree | Neutral | Agree | Strongly Agree |
| --- | --- | --- | --- | --- | --- |
| Chest x-ray |  |  |  |  |  |
| Pelvic x-ray |  |  |  |  |  |
| Musculoskeletal x-ray (i.e. shoulder, elbow, wrist, hand, knee, ankle, foot, etc.) |  |  |  |  |  |
| Abdominal x-ray |  |  |  |  |  |
| Soft tissue neck x-ray (i.e. pediatric stridor) |  |  |  |  |  |
| X-ray for line or tube placement (central line, endotracheal tube, nasogastric/gastric tube) |  |  |  |  |  |
| CT brain (non-contrast) |  |  |  |  |  |
| CT cervical spine |  |  |  |  |  |
| CT/CT angiography brain and neck (i.e. stroke protocol) |  |  |  |  |  |
| CT chest |  |  |  |  |  |
| CT angiography chest (i.e. PE) |  |  |  |  |  |
| CT abdomen/pelvis |  |  |  |  |  |
| CT extremity |  |  |  |  |  |
| MRI brain |  |  |  |  |  |
| MRI spine |  |  |  |  |  |

1. Please use the following space to elaborate on any of your responses or share any additional comments.
   1. Free Text Response
2. Please select your program format?
   1. PGY 1-3 years
   2. PGY 1-4 years
   3. Other
3. Which of the following best describes your primary clinical site?
   1. County
   2. University
   3. Community
   4. Other: Please describe below: Free Text Response
4. Please select your program region.
   1. Western Region (AK, AZ, CA, CO, HI, ID, MT, NM, NV, OR, UT, WA, WY)
   2. North Central Region (IA, IL, IN, MI, MN, ND, NE, OH, SD, WI)
   3. South Central Region (AR, KS, LA, MO, OK, TX)
   4. South East Region (AL, FL, GA, KY, MS, NC, PR, SC, TN, VA, VI, WV)
   5. North East Region (CT, DC, DE, MA, MD, ME, NH, NJ, NY, PA, RI, VT)
5. How often does your clinical practice environment have radiology resident coverage?
   1. Never
   2. Rarely
   3. Sometimes
   4. Often
   5. Always
6. How often does your clinical practice environment have radiology attending coverage?
   1. Never
   2. Rarely
   3. Sometimes
   4. Often
   5. Always
